# Nutritional intervention targeting muscle-brain axis in dementia continuum: from mechanisms to practice

**DOI:** 10.3389/fnut.2026.1966218

**Published:** 2026-09-30

**Authors:** Mehmet Akif Topcuoglu, Ayse Guler, Meltem Gulhan Halil, Hasmet Hanagasi, Ipek Midi, Bulent Saka, Esen Saka Topcuoglu

**Affiliations:** 1 Department of Neurology, Hacettepe University Faculty of Medicine, Ankara, Türkiye; 2 Department of Neurology, Ege University Faculty of Medicine, İzmir, Türkiye; 3 Division of Geriatrics, Department of Internal Medicine, Hacettepe University Faculty of Medicine, Ankara, Türkiye; 4 Department of Neurology, Istanbul University Istanbul Faculty of Medicine, Istanbul, Türkiye; 5 Department of Neurology, Marmara University Faculty of Medicine, Istanbul, Türkiye; 6 Department of Internal Medicine, Istanbul University Istanbul Faculty of Medicine, Istanbul, Türkiye

**Keywords:** aging, cognitive impairment, dementia, frailty-sarcopenia, HMB-vitamin D, malnutrition, muscle-brain crosstalk, muscle-targeted nutrition

## Abstract

Rapid aging of global population has led to an attention increasingly focusing on age-related changes in muscle and cognitive functions, specifically the interplay between sarcopenia, frailty and cognitive impairment as the interconnected geriatric syndromes with major health consequences. Despite being the central pathophysiological substrate across these geriatric syndromes, skeletal muscle loss is still insufficiently prioritized as a treatment objective. Incorporating the “malnutrition-sarcopenia-frailty” triad into dementia care is essential for continuity of management, as muscle preservation is fundamental to sustaining cognitive function within an active aging paradigm. This perspective reframes longevity by shifting emphasis from anticipating frailty to living with strength, valuing added years through resilience rather than decline. This narrative review, prepared by experts in neurology and geriatrics, explores the muscle-brain crosstalk and highlights skeletal muscle loss as a modifiable target for nutritional intervention in cognitive impairment and dementia. In conclusion, given the bidirectional links between malnutrition, sarcopenia, frailty, and cognitive decline through shared aging mechanisms, a high protein oral nutritional supplement [enriched with beta-hydroxy-beta-methylbutyrate (HMB)] and vitamin D emerges as a practical, compliant strategy to support muscle health and potentially contribute to preservation of cognitive health in older adults with or at risk of dementia, although direct evidence for cognitive benefit remains limited.

## Introduction

Global aging trends reveal rapid growth of older populations, particularly the 80+ age group (1, 2). The number of people aged 60+ is expected to double from 1 billion in 2020 to 2.1 billion by 2050 with thrice as many persons aged 80+ (1).

Aging is the strongest risk factor for most neurodegenerative disorders, as the leading cause of disability and the second-most common cause of mortality after cardiovascular diseases (3, 4). Dementia is widely recognized as one of the most overwhelming public health challenges in the aging population (5, 6). Sarcopenia and frailty are also concurrently prevalent in the elderly, especially in those with neurological disorders including dementia (2, 7, 8).

Cognitive impairment spans a continuum from mild cognitive impairment (MCI) to the dementia which is the most severe form of cognitive impairment (5, 9–11). Dementia is characterized by a decline in memory plus at least one of other cognitive domains (i.e., language, visuospatial, or executive function) (5, 9–11). MCI is considered a stage between age-related cognitive decline and neurodegenerative dementia (5, 11, 12). However, dementia is a devastating clinical syndrome encompassing many progressive and acquired neurocognitive disorders such as Alzheimer's disease, Lewy-body dementia, vascular dementia and mixed forms (5, 11, 12). The prevalence of MCI among individuals aged 60+ ranges from 6.4 to 25% and increases with age (13). The risk of conversion from MCI to dementia also increases progressively with age, while the global number of people living with dementia is projected to double every 20 years, as driven largely by population aging (12–14).

Sarcopenia and cognitive impairment are interrelated age-associated conditions, each exerting reciprocal effects: reduced muscle strength negatively impacts cognitive performance, while cognitive decline impairs muscle function (15, 16). Thus, the prevalence of cognitive impairment is higher in people with sarcopenia (54.4 vs. 20.9% in those without sarcopenia), while sarcopenia is also more prevalent in patients with dementia (26.4 vs. 9.3% in those without dementia) (15, 17, 18). Global aging has intensified attention on age-related changes in muscle and cognitive functions, specifically the interplay between sarcopenia, frailty and cognitive impairment as the interconnected geriatric syndromes with profound health implications (6, 15, 19, 20).

Skeletal muscle loss seems to the common denominator in these geriatric syndromes, where reduced muscle mass/strength (sarcopenia) increases vulnerability to physical weakness (frailty) and cognitive impairment, often mediated by malnutrition, chronic inflammation and shared biological pathways (15, 19–22).

Besides, elderly adults living with these syndromes often experience anabolic resistance, a blunted response of muscle protein synthesis (MPS) to standard nutritional stimuli, which accelerates muscle loss and limits the effectiveness of conventional nutritional strategies (21–23).

Hence, targeted nutritional interventions that can ameliorate, or even preserve, loss of muscle mass and strength, thereby improving functionality, are considered a key clinical priority and an urgent task to improve patient outcomes in the field of geriatric medicine and neurology (7, 21–26).

However, despite common risk factors and shared mechanisms of sarcopenia, frailty and cognitive impairment, the importance of muscle health as a therapeutic target in these geriatric syndromes is still underappreciated with a persistent gap between research findings and clinical practice (21, 22, 27).

This expert review seeks to bridge this gap by addressing the “malnutrition-sarcopenia-frailty triad” and muscle-brain axis, within a novel integrative paradigm that prioritizes skeletal muscle loss as a modifiable target for nutritional interventions aimed at mitigating cognitive decline and dementia.

## “Sarcopenia-frailty-malnutrition” triad in aging: a vicious cycle linked to cognitive decline

### Sarcopenia

Sarcopenia is characterized by the progressive, age-related decline in skeletal muscle mass (~1%−2% loss per year after age 50) combined by a much faster loss of muscle strength (~1.5%−5% loss per year after age 50) and/or reduced physical performance (7, 27–31).

It affects 10%−16% of the older adults worldwide and constitutes a key component of physical frailty (27, 32–34).

The European Working Group on Sarcopenia in Older People (EWGSOP 2) prioritizes muscle strength as primary screening criterion [handgrip strength (HGS) < 27 kg in men and < 16 kg in women] (29). This distinguishes three categories of sarcopenia including probable sarcopenia (low muscle strength), confirmed sarcopenia (plus low muscle quantity/quality) and severe sarcopenia (plus low physical performance) (29). The chair stand test (CST) can be used to determine lower extremity muscle strength, with consideration of CST >15 s for five stands to be indicative of sarcopenia. Traditional bioelectrical impedance analysis (BIA) method, however, remains widely used, which is based on the sum of appendicular skeletal muscle mass (ASM) in both arms and legs divided by the height in meters squared (ASM/height^2^ ≤ 7.0 kg/m^2^ for men and ≤ 5.5 kg/m^2^ for women) (20, 29).

Physical performance measures such as gait speed ( ≤ 0.8 m/s), short physical performance battery (SPPB ≤ 8-point score), timed-up and go test (TUG ≥ 20 s), or the 400 m walk test (≥6 min for completion) can be applied to determine muscle functionality and thus the severity of sarcopenia (29).

Although considered to be primarily an age-related condition, sarcopenia can also develop secondary to other predisposing factors, given its close associations with malnutrition, dementia and accelerated cognitive decline through a shared pathophysiology (6, 7, 21, 34, 35).

As a major determinant of late-life disability, sarcopenia is associated with a wide range of adverse health outcomes such as deterioration in overall functional autonomy and quality of life (QoL), higher risk of hospitalization, a prolonged hospital stay, and increased risk of mortality (7, 36, 37).

### Frailty

Frailty is defined as a geriatric syndrome characterized by a reduced biological reserve and ability to cope with endogenous and exogenous stressors with impairments across multiple physiological systems, resulting in susceptibility and vulnerability to adverse health-related outcomes (disability, death, and hospitalization) in the presence of stressors (20, 38–40).

With an approximate prevalence of 10% after age 65, frailty provides a biological framework for age-related vulnerabilities and chronic comorbidities, serving a prognostic factor for mortality and incident disability (41, 42). Although frailty is a multidimensional syndrome embracing not only physical but also psychosocial and cognitive domains, the diagnosis is primarily based on the physical frailty phenotype, known as the Fried criteria (20, 38, 43). Fried criteria include parameters of unintentional weight loss, self-reported exhaustion, weakness, slow walking speed, and low level of physical activity (≥3 criteria: frailty; 1–2 criteria: pre-frailty) (20, 38, 43).

Despite the organ-specific nature of sarcopenia and the whole-body nature of frailty, there is overlap with respect to their definitions and diagnosis, particularly in terms of the muscle decline, including reductions in muscle mass, strength and performance (44, 45). Sarcopenia is considered a key biological substrate of physical frailty and is also implicated in cognitive frailty (7, 20, 27, 46–48). Furthermore, many age-related processes leading to frailty contribute simultaneously to brain aging and cognitive impairment (43, 49, 50). This suggests the presence of shared mechanisms often creating a vicious cycle that accelerates disability, loss of independence and mortality in older adults (43, 46–50).

Figure 1 illustrates a conceptual framework of frailty and sarcopenia based on overlapping and distinct components of each geriatric syndrome.

**Figure 1:**
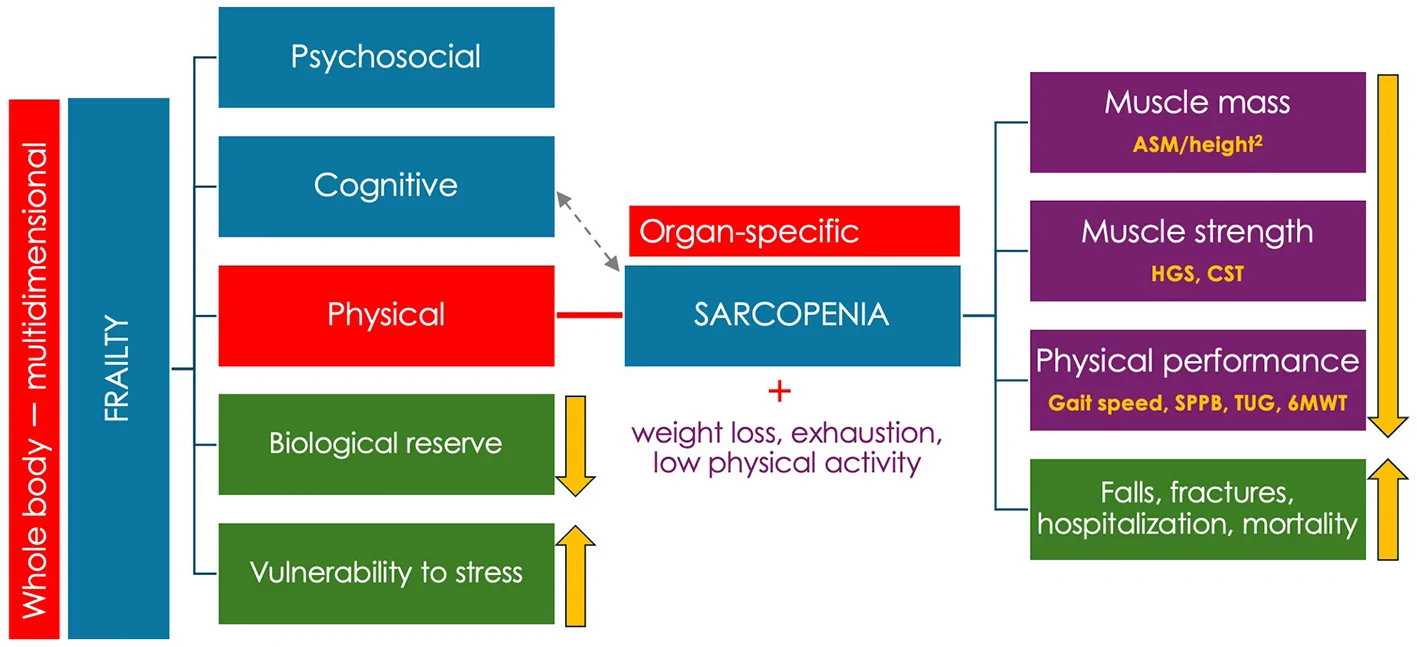
A conceptual framework of frailty and sarcopenia, based on established human evidence and experimental/preclinical mechanisms. ASM, appendicular muscle mass; CST, chair-stand test; HGS, hand grip strength; 6MWT, 6-min walk test; SPPB, short physical performance battery; TUG, timed-up and go test.

### Interplay between sarcopenia, frailty and malnutrition in the setting of aging and dementia

Sarcopenia and frailty frequently overlap with malnutrition among older adults, which is largely driven by the anorexia of aging and progressive deterioration in skeletal muscle composition and function. This convergence further increases the risk of adverse health outcomes, including mortality (20, 21, 51–53). Both malnutrition and sarcopenia are highly prevalent in neurodegenerative diseases or disorders associated with immobilizing disability, particularly the stroke and dementia (8, 22). Malnutrition typically precedes and aggravates sarcopenia, while its prevalence increases as neurological disorders progress to advanced stages. In the setting of aging, their coexistence further diminishes physical performance and QoL, often leading to physical frailty and elevating the risk of morbidity and mortality (8, 22, 24, 54). In fact, given the interaction between nutrition and neurological status, malnutrition is considered both as the cause and consequence of certain neurological pathologies (22, 55). The many pathophysiological changes associated with dementia (i.e., loss of smell, taste and appetite, swallowing disorders, disorders of orientation and attention, loss of eating and drinking skills, social isolation) cause decreased food and fluid intake and deterioration of nutrition and hydration status, which itself contributes to acceleration of dementia (24, 56, 57). Overall, the pathophysiology of sarcopenia and frailty is inextricably involved with aging and malnutrition, while there are also other common contributors including oxidative stress, mitochondrial dysfunction, systemic inflammation, vitamin D deficiency, hormonal changes and dysregulation of protein metabolism. They all at once interact to compromise neuromuscular integrity, functional capacity and metabolic flexibility (7, 20, 21, 34, 43, 58–61). These processes provide a plausible biological framework for the *malnutrition-sarcopenia–frailty* triad in aging, establishing a self-perpetuating cycle that contributes to cognitive decline. Together, they represent an interconnected multisystem syndrome rather than distinct disease entities (7, 15, 20, 27, 42, 43, 46, 53, 58, 59) (Figure 2).

**Figure 2:**
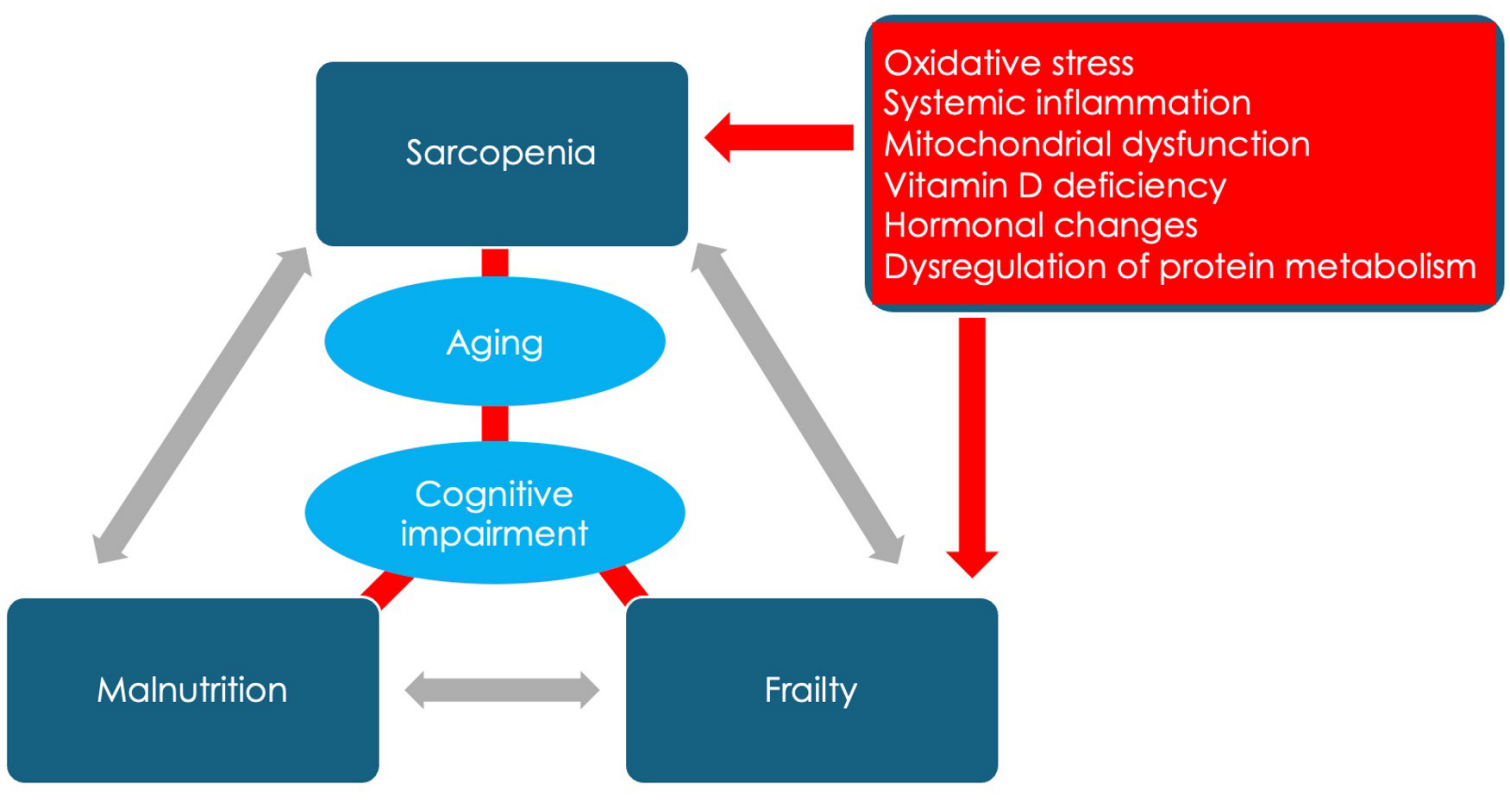
The “malnutrition-sarcopenia-frailty” triad in aging, forming a vicious cycle linked to cognitive decline, based on established human evidence and experimental/preclinical mechanisms.

### Bi-directional relation of cognitive impairment with both sarcopenia and frailty: evidence from cross-sectional and longitudinal studies

Accumulating evidence from a series of cross-sectional and longitudinal studies suggest that declining muscle strength and functionality accelerates cognitive deterioration and vice versa in older adults, emphasizing a mutual relationship (15, 18, 27, 62). It has been consistently reported that sarcopenia increases the risk of cognitive decline and dementia which in turn affects muscle function and increases the risk of sarcopenia (6, 15, 16, 48, 62–65). The interplay between the skeletal muscle and the brain, acting through the links between sarcopenic traits (low muscular strength and quality) and cognitive decline (cortical thickness, brain volumes and white matter microstructure), is considered likely to be relevant for the development of dementia (35, 46).

Similarly, frailty and cognition are tightly interconnected. Community-dwelling older adults with pre-frailty and frailty exhibit an increased risk of developing cognitive impairment compared to robust peers, while cognitive impairment itself is significantly associated with frailty progression (43, 50, 66–68). This reciprocal relationship between physical and cognitive frailty is underscored by the parallel worsening of physical disability and cognitive decline in dementia patients, and by the inverse correlation between Fried criteria and modified mini-mental state examination (MMSE) scores, indicating strong associations between higher levels of frailty and lower levels of cognitive function (43, 50, 66).

## Physiological changes in sarcopenic/aging skeletal muscle: potential connections to cognitive decline

Sarcopenia is associated with changes in skeletal muscle physiology at the metabolic, cellular, vascular, and inflammatory levels, including (a) age-dependent decline in skeletal muscle mass, strength and quality, (b) neuromuscular junction (NMJ) and motor unit degeneration and (c) mitochondrial dysfunction along with inflammatory and catabolic signaling dominance. These changes collectively lead to muscle architecture remodeling, muscle atrophy, decline in contractile activity, and impaired regeneration and anabolic resistance (7, 42, 63, 69–74) (Figure 3).

**Figure 3:**
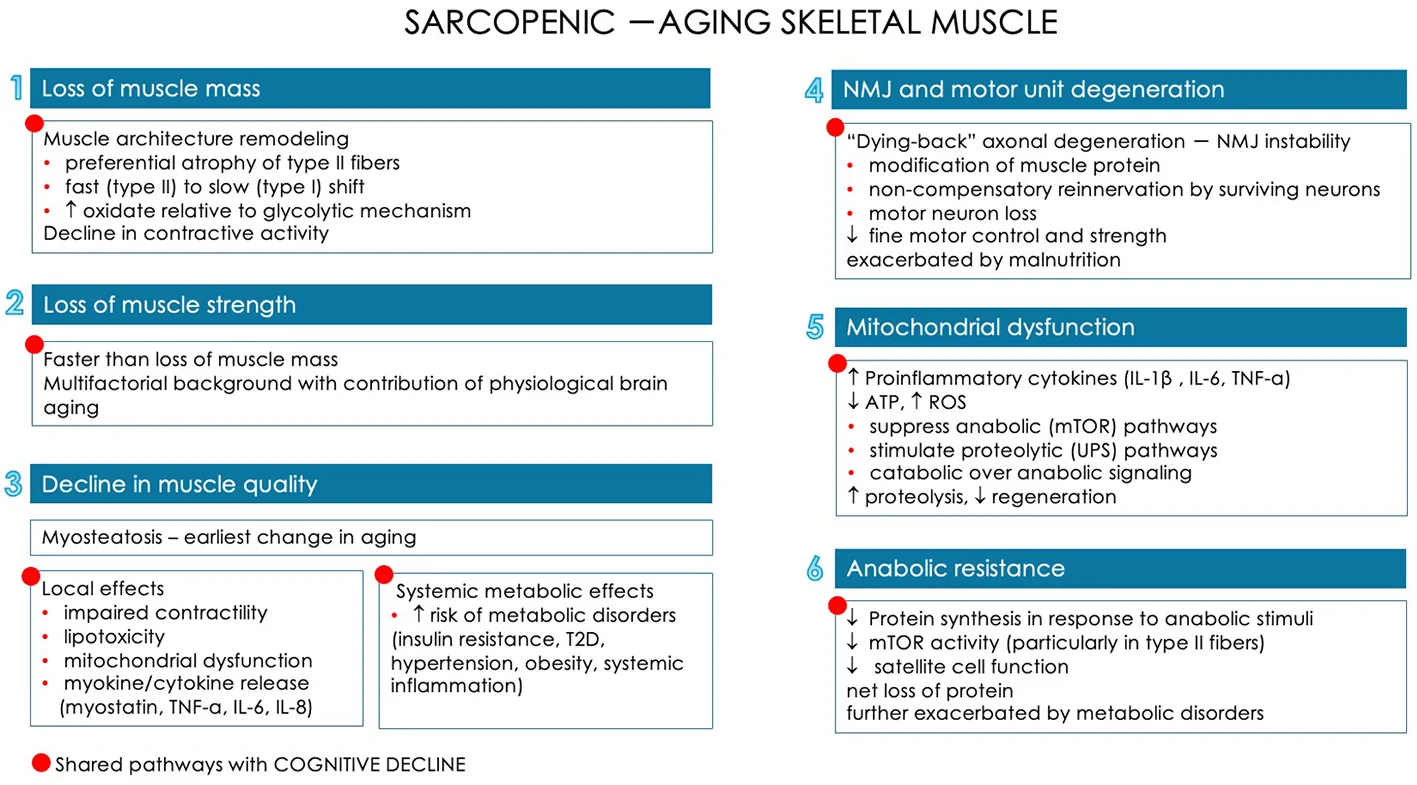
Physiological changes in sarcopenic-aging skeletal muscle with connections to physiological brain aging and cognitive decline, based on established human evidence and experimental/preclinical mechanisms. HGS, handgrip strength; IL, interleukin; mTOR, mechanistic target of rapamycin; ROS, reactive oxygen species; T2D, type 2 diabetes; UPS, ubiquitin–proteasome system.

### Age-dependent decline in skeletal muscle mass, strength and quality

Skeletal muscle mass loss with aging is caused by atrophy of muscle fibers and a decrease in the number of myofibers, mainly due to a progressive degeneration of motor neurons. This neurogenic process leads to muscle architecture remodeling and decline in contractile capacity (42, 71, 72, 74, 75). During skeletal muscle aging, the loss of fast-twitch type II fibers (with high glycolytic capacity) occurs earlier than the loss of slow-twitch type I fibers (with higher mitochondrial content and oxidative capacity) (42, 70, 76).

This preferential atrophy of type II fibers, as driven by the selective denervation of fast motor units and preferential collateral reinnervation of slow fibers, causes a net shift from fast to slow muscle fiber composition (75, 77, 78). This transition enhances oxidative metabolism relative to glycolytic activity, favoring endurance but reducing the maximal power output (75, 77, 78). Strength and resistance training targets activation of motor units, particularly type II muscle fibers, ultimately increasing muscle strength and power. However, aerobic exercise primarily improves cardiovascular fitness and targets type I muscle fibers to enhance substrate metabolism. Therefore, strength and resistance training, rather than the aerobic exercise, has been associated with improved cognitive function in older adults (71, 79, 80).

Age-related loss of muscle strength progresses 2–5 times faster than the loss of muscle mass, emphasizing the contribution of multiple factors (i.e., neural factors) besides the muscular factors in the process of decline in muscle strength (81, 82).

Age-related decline in muscle quality, characterized by progressive myosteatosis (insidious intramuscular fat infiltration) is one of the earliest structural alterations in skeletal muscle aging (71, 83). In myosteatosis, ectopic fat infiltration disrupts the muscle microenvironment via lipotoxicity, mitochondrial dysfunction, impaired contractility, and proinflammatory cytokine release (71, 83). As a result, muscle behaves as a dysregulated endocrine organ, overproducing and secreting catabolic myokines such as myostatin (71, 83). Hence, myosteatosis has substantial effects both locally on muscle function (muscle contractility performance) and systemically on the metabolism increasing the risk of metabolic disorders (i.e., insulin resistance, type 2 diabetes, hypertension, obesity and systemic inflammation) (71, 84, 85).

These factors (inflammation, oxidative stress and metabolic dysregulation), in turn, have well-established causal relationships with cognition and dementia in older adults, emphasizing that muscle quality, as measured by myosteatosis, may be strongly associated with brain health (35, 71, 86, 87).

### NMJ and motor unit degeneration

At the peripheral level, sarcopenia is strongly influenced by age-related degeneration of motor neurons (dying-back axonal degeneration) and progressive instability of the NMJ (72, 74). Weakened mechano-transduction and reduced contractile efficiency due to limited motor unit recruitment, as combined with the inadequate compensatory reinnervation by surviving motor neurons, are considered responsible for eventual muscle atrophy (72–74). This cascade initiates a self-reinforcing vicious cycle which may contribute to cognitive impairment through decreased social stimulation and low cognitive engagement with reductions in physical performance and physical activity (71, 88).

### Proteostatic imbalance, impaired regeneration, and anabolic resistance

Aging is associated with a decrease in the number and responsiveness of satellite cells, remodeling of extracellular matrix (i.e., fibrosis and increased stiffness) physically obstructing the reinnervation of denervated fibers, and dysregulation of anabolic and catabolic signaling pathways in the skeletal muscle fibers (7, 72, 89). Aging muscle develops proteostatic imbalance, marked by the suppression of anabolic mammalian (mechanistic) target of rapamycin (mTOR) pathway responsible for protein synthesis, and the activation of protein degradation through the ubiquitin–proteasome system (UPS) (7, 76, 78, 90). Myofibrillar protein synthesis is impaired by the inability of satellite cells (especially within fast-twitch type II fibers) to react positively to growth factors, cytokines and myokines (7, 63, 69).

Hence, beyond neural degeneration, the muscle microenvironment in aging progressively loses its capacity to support reinnervation and repair, besides a progressive reduction in muscle responsiveness to anabolic stimuli (anabolic resistance), a key driver of muscle fiber atrophy (7, 69, 70, 72, 76, 90).

### Mitochondrial dysfunction, inflammatory, and catabolic signaling dominance

Sarcopenia is associated with chronic low-grade inflammation phenotype (CLIP) and oxidative stress which induce catabolic signaling to accelerate proteolysis and impair regeneration (7). The systemic increase in proinflammatory cytokines, such as interleukin (IL)-1β, IL-6, and tumor necrosis factor-α (TNF-α), results in mitochondrial dysfunction and oxidative stress which further exacerbates mitochondrial damage, besides promoting fibrosis, fatty infiltration, protein degradation and accelerated apoptosis (7, 59, 70, 76). Increased levels of pro-inflammatory cytokines suppress mTOR activity and simultaneously stimulate proteolytic pathways such as UPS, which plays a major role in muscle degradation (structural and contractile proteins) (7, 70, 91). Together, these alterations are associated with a sustained predominance of catabolic over anabolic signaling (7, 59, 90, 92).

Disturbed mTOR signaling also affects mitochondrial function, glucose metabolism, energy production, and autophagy in the aging brain, leading to mitochondrial dysfunction and oxidative stress. This may directly damage mitochondrial proteins and impair cellular energy production (70, 93, 94). Hence, age-dependent disturbance in mTOR signaling is considered a shared mechanism linking sarcopenia with frailty and cognitive decline (7, 27, 35, 46, 62, 78).

### Muscle strength: a reliable indicator of cognitive health

Muscle strength is a dynamic measure of neuromuscular function, providing insights into muscle health and functionality and overall neuromuscular integrity (19). Hence, muscle strength, as measured by HGS, is considered as a more reliable indicator of cognitive function than muscle mass indices in older adults (19, 27, 95). HGS could serve as a simple yet effective screening tool and a therapeutic target for cognitive health (19, 27, 95).

The cerebellum, as linked to other brain areas responsible for cognitive functioning (i.e., the cerebral cortex and hippocampus), is considered likely to be viewed as a messenger of cognitive decline which can be measured by physically-driven tasks such as HGS (95, 96). Decline in cerebral functioning in certain forms of dementia may impair cerebellar functioning, and thereby the motor and neural pathways responsible for performing a maximal grip force generation (95). The cognitive domains affected by muscle strength are suggested to involve frontal lobe executive functions (i.e., visuospatial and motor speed via digit symbol substitution test [DSST]) and processing speed and memory function (63, 97, 98).

As summarized in Table 1, cross-sectional studies have consistently demonstrated an association between muscle strength (HGS, in particular) and cognitive performance (97–109).

**Table 1:** Muscle strength-cognitive impairment association: evidence from cross-sectional studies.

Reported “muscle strength-cognitive impairment” association in studies		References
Community-dwelling older adults		
Aged 60+	Each 1 kg deficit in baseline HGS conferred a 1.5% greater risk of developing AD over 5.7 years (adjusted HR 0.986, 95% CI: 0.973–0.998)	(100)
	Lower HGS was associated with cognitive impairment (SPMSQ; OR 1.81, 95% CI:1.33–2.46)	(101)
	A negative correlation between muscle strength and cognitive function (OR 0.23, 95% CI: 0.07–0.75), regardless of the cognitive test (word learning and recall, animal fluency, DSST)	(102)
	Higher quadriceps strength was associated with better cognitive performance (DSST, OR 5.003, 95% CI: 2.725–7.281), indicating its relation to speed of processing and visual–spatial processing	(97)
Aged 85+	Higher MMSE scores were associated with greater HGS and isometric knee extensor strength	(104)
	HGS was associated with scores in cognitive tests for processing speed and memory	(98)
Aged 55+	Poor HGS was associated with lowest scores in all cognitive tests including delayed word recall test (β −0.14, 95% CI: −0.27 to −0.02), verbal fluency test (β −0.14, 95% CI: −0.26 to −0.02), and trail making test (β −0.15, 95% CI: −0.27 to −0.03)	(105)
Older adults with dementia		
Aged 65+	Each 8-kg decrease in HGS was associated with a 59% increased likelihood of dementia (adjusted OR 1.59, 95% CI: 1.19–2.14)	(99)
Hospitalized older adults		
Aged 70+	Lower cognitive functioning measured by 6-CIT was significantly associated with low HGS	(103)
Longitudinally followed community-dwelling older adults		
Aged 60+	Low cognitive functioning was associated with a faster decrease in HGS	(106)
	Low HGS was found to be associated with a more rapid decrease in cognitive functioning	(107)
	Low HGS was found to be associated with a higher risk of developing Alzheimer's disease	(108)
	Low HGS was associated with poor scores on measures of global cognition (β −0.80), executive function (β −0.35), verbal fluency (β −0.31), and attention (β −0.34) over time (in women)	(109)

CI, confidence interval; DSST, digit symbol substitution test; HGS, hand grip strength; HR, hazard ratio; MMSE, mini-mental state examination; OR, odds ratio; 6-CIT, six-item cognitive impairment test; SPMSQ, short portable mental status questionnaire.

Accordingly, muscle strength appeared to drive the relationship between sarcopenia and cognitive impairment, suggesting that interventions designed to improve muscle strength may also preserve brain health or delay cognitive decline in aging (19, 27, 95, 102, 110). Indeed, decline in HGS is considered a vital sign of aging closely related to multitude of clinically-relevant health outcomes (i.e., frailty, diabetes, premature mortality) besides the muscle function and cognitive impairment (19, 95, 111).

Gait speed, a measure of muscle functionality (physical performance), has also been linked to specific cognitive domains (i.e., executive function, visuospatial ability and psychomotor function) in cross-sectionals studies of older adults (27, 112–116). These studies revealed that cognitive impairment (including MCI) is associated with poorer performance on gait speed assessments such as 6-m walk speed test and computer-based analyses of gait ability (27, 112–116). Some longitudinal studies indicated that reduced gait speed in aging (particularly the fast gait speed) predicts declines in global cognition and specific domains such as attention and psychomotor speed (117–119). Others reported that general and domain-specific cognitive decline (i.e. verbal memory and executive function) also predicts annual reductions in gait speed, emphasizing a bi-directional relationship between gait performance and cognitive function (120, 121).

Few studies have examined the relationship between low muscle quality and cognitive function (35, 122–124). A positive relationship between muscle quality (isokinetic strength in relation to leg muscle mass) and cognitive function was noted in adults aged 60+ years (122). Brachioradial and tibial muscle density (a surrogate measure of muscle quality) on peripheral quantitative computed tomography (pQCT) was associated with psychomotor function and visual learning in men aged 60–95 years (123). Higher muscle fat infiltration on whole-body magnetic resonance imaging (MRI), an indicator of poor muscle quality and a component of sarcopenia, was associated with reduced cortical thickness and lower cerebral and cerebellum gray matter volumes, diminished muscular strength and function, and increased all-cause mortality in adults (35, 124).

### Muscle-brain interactions in a wider systems-level framework of cognitive dysfunction

Indeed, an emerging metabolic hypothesis puts a new perspective on Alzheimer's disease, late-onset form in particular, redefining it as a brain expression of a systemic complex metabolic disorder, which shares pathogenic pathways with diabetes mellitus, obesity, and atherosclerosis (125). This suggests common treatment and preventive strategies for all these pathologies (125).

Hence, the muscle-brain interactions should be considered within this wider metabolic network, namely a systems-level framework, involving mitochondrial dysfunction, oxidative stress, impaired energy metabolism, inflammation, amino-acid metabolism, and systemic metabolic abnormalities (125). Accordingly, while mitochondrial dysfunction, oxidative stress, systemic inflammation, insulin resistance, and altered protein metabolism act as mechanisms connecting sarcopenia with cognitive decline, these processes are not uniquely muscle-derived but belong to a broader systemic metabolic phenotype associated with aging and neurodegeneration (125, 126).

## Muscle secrotome—Myokines: molecular messengers of muscle-brain crosstalk

The close relation between sarcopenia, cognitive decline and impaired motor coordination highlights a bidirectional muscle–brain axis (7). Although the exact neurobiological underpinnings of the sarcopenia–dementia link remains unknown, increasing evidence suggests a bidirectional muscle–brain crosstalk, based on putative molecular mechanisms linking sarcopenia to cognitive decline. These mechanisms include altered myokine secretion, mitochondrial dysfunction, inflammation, oxidative stress, insulin resistance and dysregulation of protein metabolism (7, 35, 63, 70, 73, 126–128) (Figure 4).

**Figure 4:**
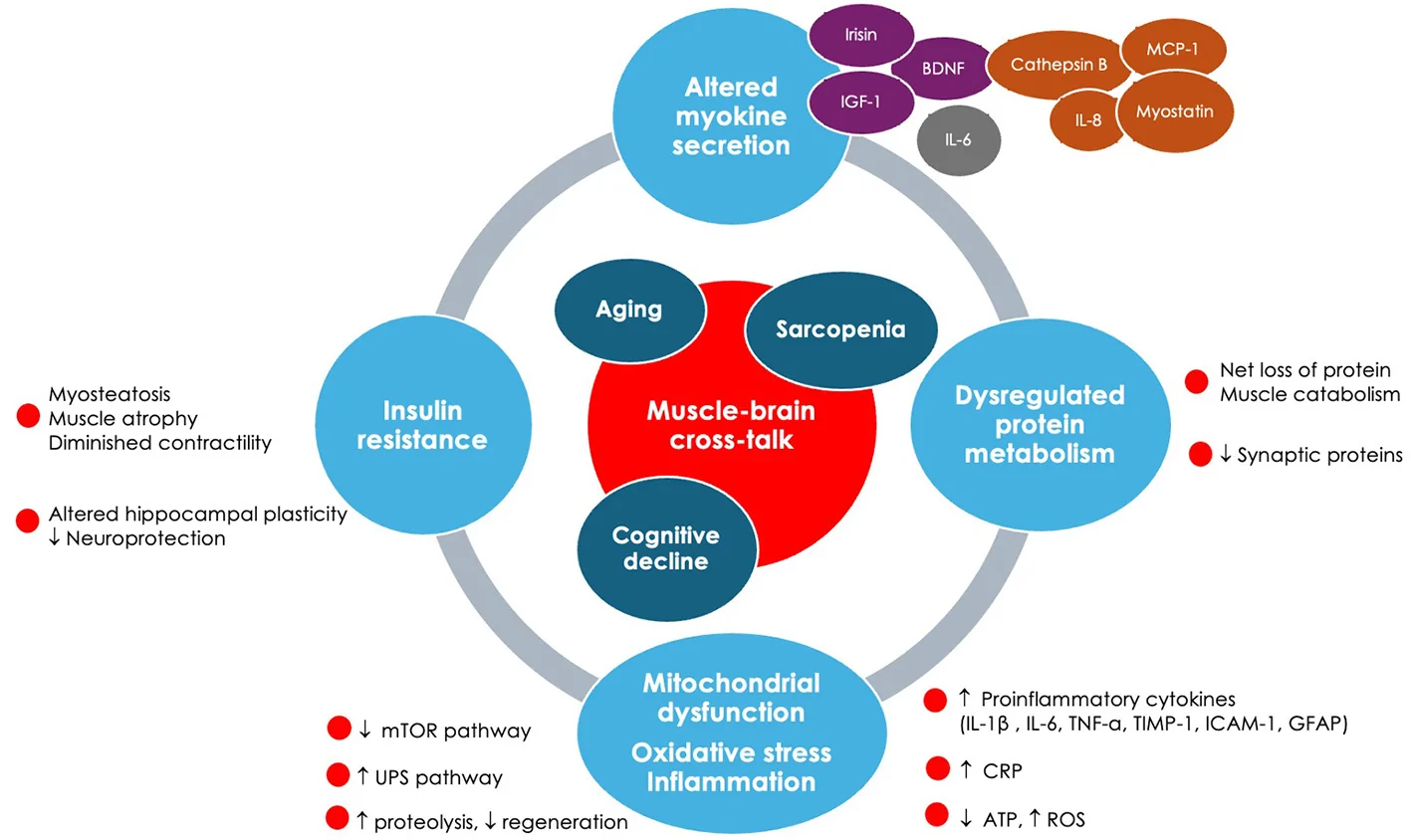
Putative pathophysiological mechanisms and conceptual framework of muscle–brain crosstalk. BDNF, brain-derived neurotrophic factor; CRP, C-reactive protein; IGF-1, insulin-like growth factor 1; IL, interleukin; mTOR, mammalian target of rapamycin; ROS, reactive oxygen species; UPS, ubiquitin–proteasome system.

Skeletal muscle is a highly active endocrine organ with a complex secretome including fatty acids, nucleic acids, and the bioactive peptides and proteins collectively termed myokines (71, 129, 130). The muscle secretome has become increasingly recognized for its systemic health effects with mediators engaging neuroplasticity, driving mitochondrial biogenesis and modulating neuroinflammatory pathways relevant to cognitive function, supporting the potential for muscle-brain crosstalk (62, 71, 129, 130). Myokines play a critical role in maintaining whole-body metabolic homeostasis through autocrine, paracrine, and endocrine signaling. They also protect and improve brain functions (i.e., cognition, memory, and motor coordination) via neurotrophic and metabolic effects (62, 63, 128–130). Hence, a complex endocrine dialogue exists between skeletal muscle and brain, which is mediated by myokines that act as molecular messengers for communication (7, 62, 63, 73, 128–131). However, age-related changes in skeletal muscle physiology (i.e., muscle atrophy with reduced regenerative capacity) adversely effects this dialogue, which may lead to altered myokine profile with hazards of weaker neuroprotection, increased inflammation and oxidative stress (7, 29, 63, 73, 128). Thus, muscle atrophy can induce neurological vulnerability and vice versa, establishing a cycle that connects sarcopenia, fragility, and neurodegeneration (7, 73, 128).

Myokines and cytokines often associated with both metabolic and brain health include brain-derived neurotrophic factor (BDNF), fibronectin type III domain-containing protein 5 (FNDC5)/irisin, insulin-like growth factor-1 (IGF-1), cathepsin B, myostatin, IL-8, monocyte chemoattractant protein-1 (MCP-1) and interleukin-6 (IL-6) (62, 71, 130).

The release of myokines is mainly mediated by transcription factors such as peroxisome proliferator-activated receptor (PPAR)-gamma coactivator-1α (PGC-1α). PGC-1α-dependent myokines include BDNF, irisin, cathepsin B, IL-6 and IGF-1. All are subsequently secreted into the circulation, and influence diverse metabolic processes (62, 71). BDNF, irisin, IGF-1 and potentially cathepsin B provides beneficial effects to both the skeletal muscle and the brain (62, 71). Locally, BDNF, irisin, IGF-1 and cathepsin B demonstrate anabolic actions on skeletal muscle. BDNF promotes myoblast differentiation, supports motor neuron survival, and functions as a contractile-inducible protein. Irisin improves metabolic dysfunction and ameliorate muscle wasting by accelerating myoblast fusion and protein synthesis. IGF-1 preserves muscle mass and function, while cathepsin B facilitates myotube formation (62, 63, 129, 131).

BDNF is a neurotrophic factor widely expressed in the central nervous system (particularly concentrated within the hippocampus and cortex), which regulates synaptic plasticity, neurogenesis, and cell survival and differentiation (132, 133). Importantly, IGF-1, FNDC5/irisin and cathepsin B are all capable of crossing the blood-brain barrier (BBB) to induce local BNDF expression, particularly in the hippocampal area. This contributes to neurogenesis, synaptogenesis and long-term potentiation (LTP) enhancement, supporting improvements in cognition and memory (62, 71, 134, 135).

Hence, BDNF is increasingly recognized as a direct mediator of muscle-brain signaling pathway and as a biomarker of both cognitive decline and resilience. Dysregulated BDNF, reductions in circulating and hippocampal levels in particular, serves as the potential mechanistic link between sarcopenia and cognitive impairment (62, 71, 132, 133, 135).

Other myokines, most notably myostatin together with proinflammatory cytokines such as IL-6, IL-8 and MCP-1 act as detrimental mediators of muscle-brain crosstalk and represent potential therapeutic targets for sarcopenia and cognitive dysfunction (62, 71, 131). Myostatin reduces muscle size and mass, drives skeletal muscle atrophy, and contributes to cognitive decline. IL-6 induces muscle atrophy, penetrates the blood–brain barrier, and accelerates cognitive decline. IL-8 promotes muscle atrophy and cognitive deterioration, while MCP-1 reduces muscle mass, disrupts blood–brain barrier selectivity to permit circulating inflammatory mediators, and hastens cognitive decline. Importantly, IL-6 exhibits a dual role. It functions as a catabolic cytokine in systemic inflammation but facilitates recovery and regeneration, particularly following exercise when secreted as a myokine from contracting muscle (62, 71, 131).

As supported by converging preclinical evidence and a growing but still incomplete body of human translational data regarding the physiological characterization, the muscle–brain axis represents a pressing biological framework with the potential of mechanistic insight embodied in the cognitive sarcopenia framework to be translated into actionable preventive clinical care for older adults (130). PGC-1α/FNDC5/BDNF pathway, chronic IL-6/IGF-1 imbalance and NMJ instability are considered the key elements of this circuit with consideration of multicomponent exercise, optimized protein/leucine intake, probiotic adjuncts, rTMS and selected mito–/inflammation-targeted pharmacotherapies as the promising interventions from the perspective of preventive geroscience (130).

## Nutritional intervention targeting muscle: a dual-benefit approach

Conventional calorie-based oral nutritional supplements (ONS) in dementia patients, although improving caloric intake and body weight, have consistently been reported to exert no beneficial effects on muscle health, cognition, or functional outcomes (8, 19, 21, 24, 42, 136, 137).

Skeletal muscle loss, a common component of both malnutrition and sarcopenia, is also a potential contributor to cognitive impairment through mechanisms of muscle-brain signaling such as inflammation and altered myokine secretion (19, 21, 70). Therefore, nutritional interventions for preserving and rebuilding muscle via molecules acting through muscle-brain crosstalk are vital for concomitant improvements in muscle health and cognitive function (19–21).

Maintaining or building muscle mass requires provision of adequate substrate for MPS (21, 138). It is well-recognized that an insufficient dietary protein intake is associated with loss of muscle mass in older adults due to lower MPS (69, 139). There is growing evidence regarding that use of high-protein ONS (HP-ONS; ≥20% of total calories as protein) can help preserve and restore muscle mass and strength in various clinical conditions associated with skeletal muscle wasting (21, 22, 140–142).

In this context, the ESPEN guidelines recently increased the protein recommendations in elderly population to 1.0–1.2 g protein/kg body weight/day for healthy older adults and to 1.2–1.5 g protein/kg body weight/day for those malnourished (or at risk of malnutrition) with acute or chronic illnesses (23). However, these weight-based targets require cautious interpretation in frail older adults with low body weight or oedema (23). The recommendations also stress the importance of high-quality proteins, particularly the branched-chain amino acids (with a focus on leucine), which are known to stimulate mTOR and IGF-1 signaling pathways to enhance MPS (7, 21, 23, 128). Beta-hydroxy-beta-methylbutyrate (HMB) and vitamin D have emerged as key molecules in muscle-targeted nutrition, given their potential to act through muscle-brain crosstalk mechanisms connecting sarcopenia and cognitive decline (7, 21, 22, 25, 35, 70, 73, 127, 128, 140, 143). Accordingly, a comprehensive muscle-targeted nutritional strategy, which aims to support anabolism and reduce catabolism in skeletal muscle, can be accomplished via combining high-protein ONS enriched with multi-nutrients such as vitamin D and HMB (a leucine-metabolite) (21, 22, 42) (Figure 5).

**Figure 5:**
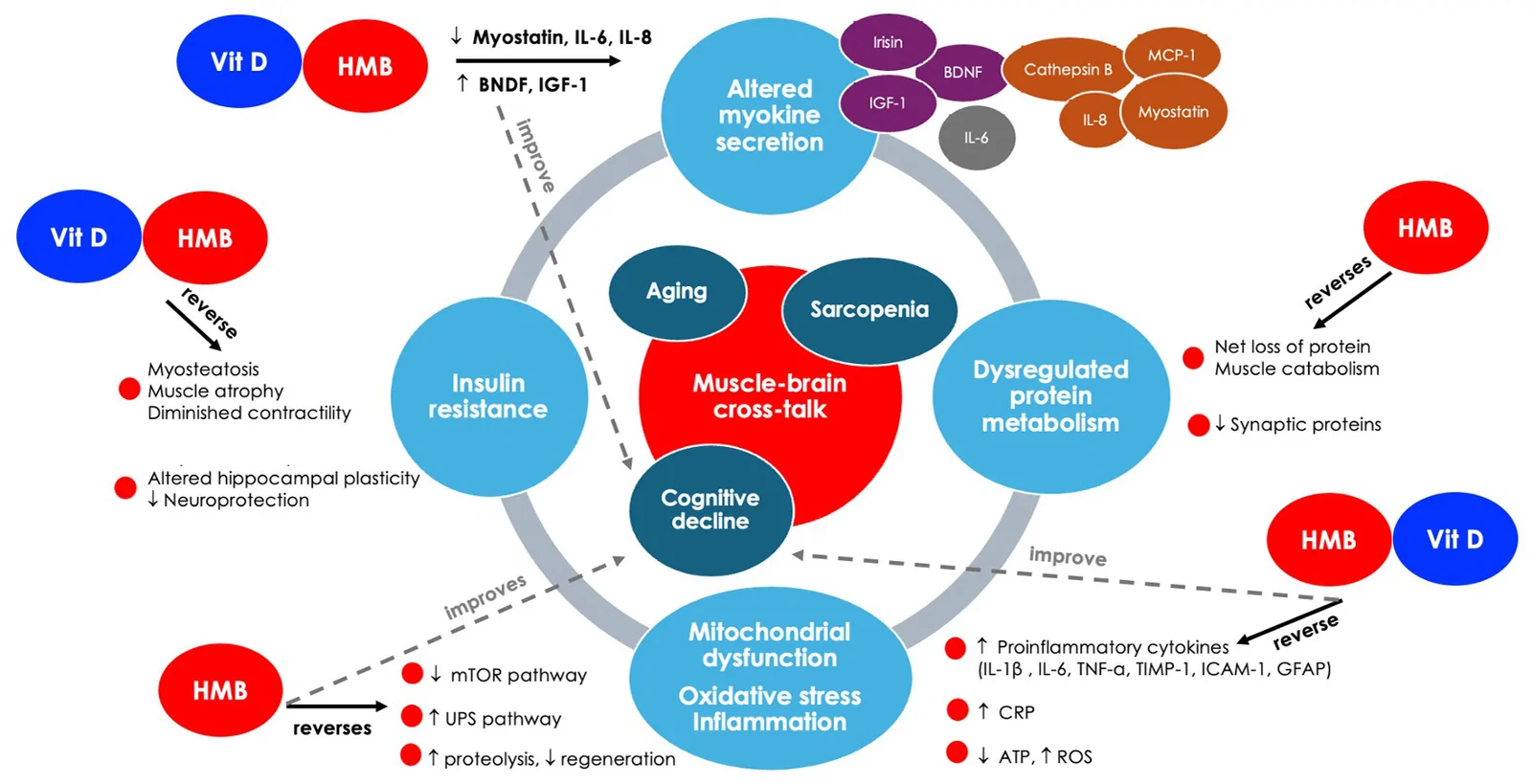
HMB and vitamin D targeting the putative pathophysiological mechanisms of muscle–brain crosstalk. BDNF, brain-derived neurotrophic factor; CRP, C-reactive protein; IGF-1, Insulin-like growth factor 1; IL, interleukin; mTOR, mammalian target of rapamycin; ROS, reactive oxygen species; UPS, ubiquitin–proteasome system.

### HMB

HMB is an active metabolite of the essential amino acid leucine which is a master dietary regulator of muscle protein turnover (22, 42). Dietary leucine does not provide a large amount of HMB, as only 5% is metabolized into HMB. However, direct ingestion of HMB more potently affects the stimulatory protein synthesis signaling, resulting in demonstrable muscle mass accretion (42, 144). Although HMB is found in certain foods (e.g., avocado, catfish, cauliflower, and grapefruit), achieving a therapeutic dose (3 g/day) is only possible through isolated supplements, either alone or in combination with amino acids such as arginine and glutamine (HMB/Arg/Gln), or via an HMB-enriched ONS (22, 25, 129).

HMB has attracted interest due to reported anabolic (a potent stimulator of protein synthesis) and anticatabolic (inhibitor of protein breakdown) effects in muscle metabolism (42, 145–147). Indeed, HMB is widely utilized by bodybuilders and power athletes as an ergogenic aid to promote exercise performance and skeletal muscle hypertrophy (148). Hence, HMB is considered to offer the additional benefit in terms of maintaining muscle mass and functional capacity in older adults (22, 145).

#### Effects on skeletal muscle

HMB acts through a dual mechanism in skeletal muscle. It enhances MPS via upregulating anabolic signaling pathways (mTOR pathway) and potentially maintaining IGF-1 signaling. Besides, it suppresses muscle protein breakdown via downregulation of catabolic signaling pathways (ubiquitin–proteasome pathway) (25, 148–150). HMB functions by preserving satellite cell function and stimulating MPS and regeneration, while it also simultaneously mitigates muscle damage and lowers pro-inflammatory markers (i.e., IL-6, IL-8), which helps to combat muscle atrophy and improve recovery capacity (151–156).

Its effects on myokine regulation are also notable, such as lowering myostatin expression (a key protein that inhibits muscle growth) to mitigate adverse effects of myostatin on muscle growth and increasing irisin expression to promote muscle hypertrophy and prevent atrophy (154, 157).

HMB supplementation may also counteract anabolic resistance, a hallmark of aging driven by oxidative stress and CLIP, as primarily regulated through the mTOR signaling pathway (25, 126, 145, 151, 158). The modulation of mitochondrial dynamics and lipid metabolism by HMB may also serve for preventing disuse atrophy and supporting rehabilitation (148).

Hence, HMB is considered an effective metabolic treatment for muscle health, contributing to the reduction of muscle damage while promoting muscle growth and recovery (148, 159). The age-related decline in the endogenous plasma HMB levels in older adults is negatively correlated with muscle mass and strength, further favoring the rationale for HMB supplementation in older adults with or at risk of muscle loss (22, 150, 160).

Table 2 summarizes the outcomes of randomized controlled trials (RCTs) regarding the effects of HMB supplementation on muscle parameters in older adults with or without sarcopenia.

**Table 2:** RCTs studying the effects of HMB supplementation on muscle parameters in older adults with or without sarcopenia.

Studies	Study population	Intervention	Control	Intervention (HMB) vs. control				HMB outweighs
				Muscle mass	Muscle strength	Muscle quality	Functionality	
Sarcopenia (+)								
Zhu et al. (161)	Adults aged 65+ with sarcopenia	ONS (8.61 g protein, 1.21 g HMB, 130 IU vitamin D and 0.29 g omega-3 fatty acid) Twice daily -12-weeks (*n* = 36)	Exercise alone (*n* = 40)	↑ (DXA)	Similar HGS	–	Similar gait speed and FTSST	Muscle mass
Nasimi et al. (162)	Adults aged 65+ with sarcopenia	300 g/d fortified yogurt (3 g/d HMB, 1,000 IU/d vitamin D, 500 mg/d vitamin C)-12-weeks (*n* = 33)	300 g plain yogurt (*n* = 33)		↑ HGS		↑ Gait speed	Muscle strength and functionality
Yang et al. (163)	Adults aged 60+ with sarcopenia	3 g CaHMB 20 kJ energy, 1.2 g carbohydrates, and 15 mg: twice daily + resistance exercise -12-weeks (*n* = 18)	Same ingredients except for HMB + resistance exercise (*n* = 16)	–	↑ HGS	↑ HGS/FFM	↑ Gait speed-and FTSST	Muscle strength, quality and functionality
Meza-Valderrama, et al. (164)	Adults aged 60+ with sarcopenia	3 g/d CaHMB; resistance exercise -12-weeks (*n* = 17)	Placebo; resistance exercise (*n* = 15)	**–**	↑ HGS (in women)	–	↑ SPPB	Muscle strength (only women) and functionality
Cramer et al. (165)	Malnourished sarcopenic patients	ONS (20 g protein; 499 IU vitamin D3; 1.5 g CaHMB) twice daily -24 weeks (*n* = 165)	ONS (14 g protein; 147 IU vitamin D3) ( *n* = 165)	Similar (DXA)	↑ Leg strength and HGS (mild-moderate sarcopenia)	↑ (leg strength relative to mass)	↑ Gait speed (mild-to-moderate sarcopenia)	Muscle strength, quality and functionality
Sarcopenia (–)								
Flakoll et al. (168)	Elderly women	2 g beta-HMB, 5 g arginine, and 1.5 g lysine daily -1 year (*n* = 27)	Placebo group (*n* = 23)		↑ HGS		↑ GUG	Muscle strength and functionality
Stout et al. (169)	Adults aged 65+ Phase I	3 g CaHMB twice daily -24 weeks (*n* = 27)	Placebo (*n* = 27)		↑ HGS	↑ HGS/arm lean mass		Muscle strength and quality
	Adults aged 65+ Phase II	3 g CaHMB twice daily + resistance training (3 d/w) -24 weeks (*n* = 27)	Placebo + resistance training (3 d/w) ( *n* = 27)	Similar (DXA)	Similar HGS	↑ Only in the HMB alone group	Similar GUG	Muscle quality
Chew et al. (166)	Adults aged 65+ at risk for malnutrition	ONS (262 kcal, 10.5 g protein, 8.5 g fat, 34.2 g carbohydrate, 7.75 mcg (310 IU) Vitamin D3, and 0.74 g calcium HMB) twice daily -6 months (*n* = 405)	Placebo 60 kcal, 1.07 g protein, 1.21 g fat and 11.9 g carbohydrate (*n* = 406)	↑ DXA (only in those with low ASMI)	↑ Leg strength ↑ HGS (in females)		Similar SPPB	Muscle mass (if low baseline ASMI) Muscle strength (leg: all patients, HGS: only women)
Rathmacher et al. (167)	Adults aged 60+ with Vit D insufficiency (*n* = 117)	HMB (3.0 g/day) plus + vitamin D3 (2,000 IU/day) (no exercise) -12 months HMB (3.0 g/day) plus + vitamin D3 (2,000 IU/day) + exercise) -12 months	Control (Ca lactate) (no exercise) control (Ca lactate) + exercise	↑ DXA (in non- exercise group)	↑ Knee extension (in non-exercise group)		↑ GUG (in non- exercise group	Muscle mass, strength and functionality (non-exercise group)

DXA, dual-energy X-ray absorptiometry; GUG, get-up-and-go; FTSST, five times sit-to-stand test; HGS, hand grip strength; HMB, β-hydroxy-β-methylbutyrate; ONS, oral nutritional supplement; PT, peak torque; SPPB, short physical performance battery.

The studies in sarcopenia patients (aged 65+) revealed that ONS-HMB (±vitamin D) supplementation significantly improved the muscle mass [assessed by dual-energy X-ray absorptiometry (DXA)] compared with exercise alone (161) or plain yogurt (161). It also enhanced muscle strength (HGS), quality [HGS/fat-free mass (FFM)] and functionality [gait speed, five-time sit-to-stand test (FTSS), SPPB] compared to resistance exercise alone (163, 164). Moreover, supplementation has been shown to improve muscle strength (HGS, leg strength) and functionality (gait speed) specifically in individuals with mild-to-moderate sarcopenia, and the muscle quality (leg strength relative to leg muscle mass) compared to ONS formulations lacking HMB (165) (Table 2).

The studies performed in community-dwelling older adults (aged 65+) without sarcopenia revealed that ONS-HMB (±vitamin D) supplementation significantly improved muscle mass, muscle strength (leg strength overall, HGS only in females) compared to placebo (166) and muscle strength and functionality in older adults with vitamin D insufficiency compared to placebo (167). Additionally, HMB/Arg/Lys supplementation was reported to improve muscle mass (DXA) and performance (GUG) significantly in women compared to placebo (168). Similarly, HMB alone significantly improved muscle strength (HGS) and muscle quality (HGS/arm lean mass) compared to placebo (169). However, when combined with exercise, HMB supplementation led to significant gains in muscle quality, but did not produce improvements in muscle mass, strength or functionality compared to exercise alone (169) (Table 2).

Meta-analyses further supported the positive effects of HMB supplementation, showing consistent improvements in the muscle mass and HGS in older adults (145, 170–174), in patients with sarcopenia (159), and in a variety of clinical conditions associated with skeletal muscle wasting (25).

In middle-aged adults, HMB was also associated with gains in muscle mass, HGS and functional performance (FTSST, gait speed) (175).

Notably, HMB/Arg/Gln combinations are considered to be more effective for enhancing muscle mass, while utilization of HMB alone or within ONS shows greater efficacy in improving muscle strength (25). A supplementation period exceeding 12 weeks is generally recommended to achieve optimal benefits (175).

Also, in a systematic review, HMB had no additional effect on body composition, strength and physical performance parameters when associated with strength training in elderly. This preliminary finding, not confirmed by other studies, suggests that the effect of HMB supplementation may be overshadowed by the strong muscle hypertrophy effect of strength training (156).

#### Effects on cognitive functions

HMB supplementation may also have potential for ameliorating age-related cognitive decline by inducing beneficial effects on memory and cognition (174, 176). Despite hydrophilic properties, HMB is able to cross the BBB and to be transported through brain endothelial cells by a proton-coupled transport system (177–179).

In experimental rat models, HMB promoted the differentiation and neurite outgrowth of Neuro2a cells (180) and prevented the age-related regression of dendritic trees in pyramidal neurons (responsible for higher cognitive processing) (175, 176, 181). It also improved working memory, behavioral flexibility, and spatial reference learning and memory tasks (correlated with hippocampal activity) and ameliorated age-related decline in cognitive performance (182, 183). Taken together, these findings suggest the likelihood of medial prefrontal cortex (mPFC) and hippocampus to be leading candidate loci for mediating HMB's effects on cognition with aging (176, 179).

Table 3 summarizes the peripheral effects of HMB on some of the physiological mechanisms in the aging brain in animal studies (93, 160, 175, 176, 179, 180, 184–190).

**Table 3:** Peripheral effects of HMB on some of the physiological mechanisms in the aging brain.

HMB affects the aging brain by:
• Saturating the rate-limiting enzyme in cholesterol synthesis which enhance neuronal synaptogenesis, specifically dendrite and synaptic terminal differentiation
• Increasing protein synthesis via the mTOR pathway which is associated with neural plasticity, cell growth, and cognition and known to decrease in activity within the aging brain
• Upregulating the growth hormone and/or insulin-like growth factor-1 (GH/IGF-1) axis, which seems notable given the decrease in IGF-1 and IGF-1 receptors in the aging brain as associated with cognitive decline, while IGF-1 is also crucial for dendritic growth
• Increasing cell health, possibly via mTOR-driven reduction of oxidative stress as well as through altering immune function leading to reduced expression of proinflammatory markers (i.e., TNF-a, IL-8, IL-10, interferon-γ)
• Enhancing cell survival, differentiation and protein turnover in muscle, mainly activating PI3K/Akt and mitogen-activated protein kinases/extracellular-signal-regulated kinases (MAPK/ERK) signaling pathways, which are two pathways related to neuronal survival and differentiation
• Promoting neurite outgrowth in mouse neuroblastoma Neuro2a cells, as mediated by activation of both the PI3K/Akt and ERK1/2 signaling
• Promoting activity of myocyte-enhancer factor 2 family (MEF2) family of transcription factors which are involved in neuronal survival and plasticity, as regulated by PI3K/Akt and ERK1/2 signaling
• Binding to the nuclear hormone receptor PPARα in the brain, which stimulates the expression of neurotrophic factors (such as BDNF) essential for the survival, growth, and maintenance of neurons, while promoting BNDF-mediated hippocampal plasticity and density of dendritic spines which are crucial for memory

In a *post hoc* analysis of the NOURISH trial, ONS-HMB benefits were further associated with measurable improvements in QoL domains including mental health/cognition besides the vitality, social functioning, and general health during the post-hospitalization period compared to placebo (191).

HMB supplementation was also associated with improvements in working memory, fluid intelligence reaction time, and processing efficiency among active-duty air force men (192). Additionally, the combined effects of exercise and HMB supplementation produced synergistic improvements in cognitive functions as measured by the MMSE in institutionalized older adults aged 70+ (193).

HMB is generally safe and well-tolerated with no significant side effects when taken at standard doses (usually 3 g daily). Human studies show no harmful impacts on liver or kidney function. However, some users may occasionally experience mild stomach discomfort, such as nausea or cramps. As with any supplement, it is best to consult a doctor before use.

Nonetheless, the beneficial cognitive effects of HMB are primarily based on robust pre-clinical mechanistic signals with weak human clinical evidence derived mostly from *post hoc* analyses and secondary endpoints of trials primarily powered for physical/muscle outcomes. Hence, HMB enriched ONS may indirectly support cognition by preserving muscle health, rather than having proven direct cognitive protective effects in large scale human RCTs.

### Vitamin D

Vitamin D regulates and modulates the physiology and function of multiple human systems, inclusive of immune system, nervous system and musculoskeletal system. In immune system, it downregulates the expression of pro-inflammatory cytokines such as IL-6. In nervous system, it affects neuronal differentiation, maturation and growth, neuroplasticity and neurotransmission. In musculoskeletal system it increases the proliferation, differentiation and growth of muscle fibers (194–197). Older people are particularly prone to develop vitamin D insufficiency/deficiency and thus are at increased risk of consequent sarcopenia, frailty and falls, given that vitamin D status is independently related to both loss of muscle mass and decline in muscle strength (195, 197).

#### Effects on skeletal muscle

Vitamin D binds to the VDR receptor on muscle fibers, which is more expressed in satellite cells than in mature myofibers. Thereby, it promotes an increase of the cross-sectional area of skeletal muscle fibers, which is a key step for myogenic initiation (195, 196, 198, 199). Consequently, vitamin D stimulates the proliferation and differentiation of skeletal muscle fibers, maintaining and improving muscle strength and functionality (195). Moreover, it suppresses myostatin expression to prevent muscular degeneration and improve contractile filaments and muscle strength (195, 200). Aging is associated with progressive decline in VDR expression along with a lower functional response to vitamin D, leading to decreases muscle strength and gait speed. This emphasizes the likelihood of vitamin D to be a molecule potentially targeting the hallmarks of the aging in skeletal muscle physiology (195, 201, 202).

Muscle biopsies in patients with vitamin D deficiency revealed predominant atrophy of type II muscle fibers, intramuscular fat and glycogen infiltration, enlargement of the inter-fibrillary gaps and increased fibrosis (195, 203). Vitamin D supplementation increases both the number and the diameter of type II fibers by promoting MPS and myogenesis eventually leading to improvements in muscle strength and neuromuscular performance (204, 205). Given that cellular changes in sarcopenic muscle are also characterized by the preferential atrophy of type II fibers and the myosteatosis, reversing age-dependent decline in vitamin D levels by supplementation is considered a promising therapeutic strategy in sarcopenia (20, 22, 206).

The link between vitamin D deficiency and muscle dysfunction is considered likely to be due to the loss of vitamin D receptor function, oxidative stress and impaired mitochondrial function and increased proinflammatory cytokines like IL-8, IL-6, and TNF-α (126, 196, 207).

Several observational studies have highlighted the role of vitamin D supplementation in preserving or improving muscle strength and function, thereby supporting the biological link between a low vitamin D level and the age-related decline in muscle mass and functionality (195, 208, 209). In a prospective study in men aged 85+, low circulating vitamin D levels were linked to accelerated loss of muscle strength (HGS), but not with physical performance (TUG) over time (208). An observational cohort study further demonstrated that daily vitamin D supplementation (20 μg for a 6 month) in post-menopausal women with osteoporosis and/or vitamin D deficiency (< 30 ng/mL) was significantly correlated to an increase of appendicular muscle strength (HGS and knee extension strength) and functionality (SPPB and 4-m gait speed), accompanied by a reduction in the risk of falls (209).

Despite extensive investigation, a clear consensus on the association between vitamin D status and muscle mass or strength has not yet been established. As outlined in Table 4, the RCTs that specifically investigated the effect of oral vitamin D supplementation on muscle health across different patient populations (baseline vitamin D insufficiency/deficiency, sarcopenia, community dwelling) have produced inconsistent findings (210–219). Certain studies indicated improvement in muscle strength (210, 213) and functionality (210, 213) in community-dwelling or institutionalized elderly people as well as improvement in muscle mass (216, 217) and functionality (217) in those with pre-sarcopenia, sarcopenia or frailty.

**Table 4:** RCTs on the effect of vitamin D supplementation for the prevention of sarcopenia and frailty.

Studies	Population	Intervention	Control	Intervention (Vit D) vs. placebo/control			Vit D outweighs
				Muscle mass	Muscle strength	Functionality	
Pfeifer et al. (210)	Community-dwelling adults aged 70+ with Vit D ≤ 78 nmol/L (*n* = 242)	1,000 mg calcium/day + 800 IU Vit D/day −12 months	1,000 mg calcium/day	–	↑ HGS, knee extension strength	↑ SPPB, TUG and gait speed	Muscle strength and functionality
Shea et al. (211)	Community-dwelling adults aged 60+ with Vit D ≤ 20 ng/mL (*n* = 100)	858 (+800) IU Vit D/day −1 year	Placebo/day	No improvement	–	No improvement (backward tandem walk test, SPPB)	None (mass, strength)
Aschauer et al. (212)	Older adults (aged 65–85 years) ( *n* = 100)	800 IU vitamin D3 per day, 50,000 IU vitamin D3 per month 400 mg of calcium daily + resistance training −16 weeks	Control (resistance training)	–	No improvement in CST	No improvement (gait speed, TUG, chair stand test)	None (muscle functionality)
Qi et al. (213)	Middle-aged and elderly individuals (*n* = 160)	Vit D supplementation + health education and lifestyle guidance −3 months	Control (health education and lifestyle guidance)		↑ HGS, pinch strength, CST	↑ TUG	Muscle mass and functionality
Haghighi et al. (214)	Postmenopausal women (*n* = 44)	Vit D (50,000 IU every 2 weeks) Resistance training +Vit D −12 weeks	Placebo Resistance training + placebo	–	No further improvement (upper and lower limb)	–	None (muscle strength)
Uusi-Rasi et al. (215)	Community-dwelling women aged 70–80 years with fall history (*n* = 409)	800 IU vitamin D/day ± exercise −2 years	Placebo/day ± exercise	No improvement	–	No improvement (gait speed, SPPB, TUG, FTSST)	None (mass and functionality)
El Hajj et al. (216)	Pre-sarcopenic elderly people (*n* = 128)	Vit D (10,000 IU) 3 × /week −6 months	Placebo	↑ ASM	No improvement	–	Muscle mass
Verlaan et al. (217)	Sarcopenic older adults (*n* = 380)	20 g protein + 3 g fat + 9 g carbohydrates + 800 IU Vit D twice daily −13 weeks	Iso-caloric control product twice daily	↑ BIA (affected by baseline Vit D and protein intake)	↑ CST (not affected by baseline Vit D and protein intake)	–	Muscle mass and functionality
Latham et al. (218)	Frail older people (*n* = 243)	Single dose of 300.000 IU −10 weeks	Placebo (single dose)	–	–	No improvement (quadriceps resistance exercise)	None (functionality)
Levis et al. (219)	Sedentary men aged 65+ with Vit D < 30 ng/mL and SPPB ≤ 9 (*n* = 130)	4.000 IU Vit D/day −9 months	Placebo/day	–	–	No improvement (SPPB, gait speed	No differences in SPPB, gait speed, falls

ASM, appendicular skeletal muscle mass; BIA, bioelectrical impedance analysis; CST, chair-stand test; DXA, dual-energy X-ray absorptiometry; FTSST, five times sit-to-stand test; HGS, hand grip strength; SPPB, short physical performance battery; TUG, timed up and go; Vit D, vitamin D.

Evidence from systematic reviews and a meta-analysis suggests a significant positive effect of vitamin D supplementation on muscle mass [standardized mean difference (SMD) 0.27] and strength (SMD 0.34) but no effect on muscle functionality (gait speed) in community-dwelling older adults (220, 221). Conversely, some studies revealed no significant effect of vitamin D supplementation on muscle strength in community-dwelling older adults (222, 223). In patients with sarcopenia, supplementation was linked to significantly improved muscle strength (HGS), while muscle mass or functionality (sit-to-stand time) remained unchanged (224).

The SHIELD study reported a significant improvement in vitamin D status in the intervention (ONS containing HMB + Vit D) group, as compared to worsening vitamin D status in the control group (166). Vitamin D deficiency in older people is suggested to be effectively addressed by ONS interventions, which is expected to help avert the adverse outcomes such as increased risk of functional impairment, frailty, falls, and mortality (166, 225).

In a review summarizing data from 44 studies (13 RCTs, 13 animal dietary intervention and 18 experimental studies) regarding the effects of vitamin D on skeletal muscle, it was concluded that at cell and animal levels, vitamin D supplementation had positive effects on the development of muscle fibers in cells in culture, skeletal muscle force and hypertrophy, besides its regulatory effects on lipid, glucose and mitochondrial muscle metabolism (205). However, when viewed from the clinical perspective, the evidence largely indicates a predominance of null effects of vitamin D supplementation on muscle health outcomes in the aging population, reflecting limited and inconsistent benefits across trials (205).

Indeed, while there is some evidence that suggests a potential benefit of vitamin D in sarcopenia, it remains uncertain whether dose, dosing frequency or treatment duration impacts the efficacy of vitamin D on improving muscle mass or function (22, 226). Nonetheless, a longer treatment period or a higher dose of vitamin D may be necessary in older people with baseline vitamin D deficiency to reach target vitamin D concentration and observe beneficial effects on the muscle (195, 218).

#### Effects on cognitive functions

Vitamin D receptors (VDRs) are found abundantly throughout the brain, where they promote neuroprotection and exert anti-inflammatory actions (227). Evidence suggests that vitamin D regulates brain neurotrophins, particularly by boosting hippocampal BDNF, which may attenuate age-related cognitive decline and improve mental health (228, 229). In contrast, low vitamin D levels are increasingly recognized as a predisposing factor for cognitive decline, in addition to higher vulnerability to dementia and Alzheimer's disease (230, 231).

Vitamin D supplementation (≈800 IU daily) has been shown to potentially mitigate cognitive impairment, particularly among older adults with pre-existing deficiency or MCI (232).

Only a few RCTs investigated the potential role of vitamin D supplementation in the prevention of cognitive decline (233–235). Two recently published RCTs with identical protocol (receiving 800 IU/day of vitamin D vs. starch granules as placebo) revealed the association of 12-month vitamin D supplementation (vs. placebo) with significantly improved plasma amyloid β-related biomarker levels and cognitive tests in patients with Alzheimer's disease (233), and with improved cognitive function via reducing oxidative stress (expressed as increased telomere length) in older adults with MCI (234). In contrast, the largest randomized, placebo-controlled trial of vitamin D plus calcium therapy in 4,143 women (aged ≥65 years) without probable dementia at baseline found no difference in the incidence of cognitive decline in the treatment group compared to placebo during a follow-up for 7.8 years (235).

In a multicenter study with 1,900 adults age 70+ receiving vitamin D supplementation, omega-3s, and a strength-training exercise program (alone or in combination), vitamin D did not improve cognitive function over a 3-year follow-up (236). Similarly, in older adults with mild to moderate vitamin D deficiency, 24-month daily vitamin D supplementation (vs. placebo) conferred no significant benefit for executive function or overall cognition (237). A systematic review of four trials likewise reported inconsistent and inconclusive results on the impact of vitamin D supplementation on cognition (238).

The utilization of vitamin D supplementation warrants further investigation, both for its potential role in preventing cognitive decline among healthy elderly population and for its possible benefits in improving cognition in individuals with dementia (239).

At standard therapeutic dosages, vitamin D is highly biocompatible and rarely elicits adverse effects, though mild gastrointestinal symptoms like transient constipation or nausea occasionally occur in hypersensitive patients. Conversely, excessive chronic ingestion triggers hypervitaminosis D, inducing hypercalcemia that can cause polyuria, nephrocalcinosis, and renal impairment.

#### Controversial findings and heterogeneity between studies

While observational studies consistently link low vitamin D levels to a higher risk of cognitive decline and Alzheimer's disease, interventional trials fail to show that adding supplements slows or prevents cognitive deterioration (240, 241). RCTs and meta-analyses largely confirm that vitamin D supplementation yields null, mixed, or inconsistent clinical benefits for muscle mass, physical performance, and cognitive function in general populations (242–244).

Possible sources of this heterogeneity are suggested to include baseline vitamin D status, dose, treatment duration, participant age, presence of sarcopenia/MCI, and interaction with exercise status (245, 246). High vitamin D levels in observational studies often reflect an overall healthy, active lifestyle rather than a direct protective agent (241, 245, 246). Clinical benefits of supplementation are generally limited to correcting severe, pathological deficiencies rather than optimizing already adequate blood levels (241, 245, 246).

Hence, vitamin D supplementation cannot be universally recommended for all older adults, and benefit is most probable in participants with confirmed vitamin D insufficiency or deficiency.

### High-protein ONS enriched with HMB and vitamin D: a convenient and compliant nutritional strategy targeting both muscle and brain health

Plasma free amino acid (PFAA), mainly the decreases in the levels of three branched-chain amino acids (BCAAs; valine, leucine, and isoleucine) and histidine, is associated with Alzheimer's disease development (247, 248). Population-based studies indicate that the risk of dementia is significantly reduced among individuals adhering to a diet with a high percentage of caloric intake from protein sources (249–252). Every 5% of calories coming from animal protein instead of carbohydrates corresponds to a reduction in the dementia risk by 11%. Furthermore, for every 5% of calories from plant protein instead of carbohydrates, there is a 26% reduction in the risk of dementia developing later in life (251, 252).

Preventing and reversing muscle loss via utilization of muscle-targeted nutritional interventions is considered a key clinical priority to concomitantly address malnutrition and muscle health in various patient settings (21, 22, 25, 253).

The pivotal EFFORT, NOURISH and SHIELD trials revealed significantly improved nutritional and functional outcomes with use of (a) high-protein ONS in hospitalized polymorbid patients at malnutrition risk, (b) high-protein ONS enriched with HMB elderly hospitalized malnourished patients with cardiopulmonary diseases, and (c) high-protein ONS enriched with HMB + vitamin D in community-dwelling older adults at risk of malnutrition, respectively (166, 191, 254, 255).

RCTs assessing utilization of ONS enriched with HMB + vitamin D revealed significantly improved muscle mass (161, 162) in sarcopenia patients (aged ≥ 65 years), muscle mass and strength in community-dwelling older adults (aged ≥65 years) without sarcopenia (166), and muscle strength and functionality in older adults with vitamin D insufficiency (167).

In a 24-week intervention, while both ONS formulations improved muscle strength (HGS) and functionality (gait speed), the experimental ONS with higher protein (20 g), more vitamin D3 (499 IU), and added HMB (1.5 g) produced additional gains in leg strength and muscle quality compared to the control ONS consisting of 14 g protein and 147 IU vitamin D3 (165).

Notably, in a network meta-analysis of 42 RCTs involving 3,063 elderly patients with sarcopenia, HMB was associated with improved HGS and five-time sit-to-stand test (FTSST) performance, while vitamin D was associated with improved gait speed (26). These findings were derived from comparative evaluation across seven categories of anti-inflammatory supplements including combined formulations, amino acids, whey protein, HMB, vitamin D, n-3 polyunsaturated fatty acids (PUFAs), and epicatechin (26).

Hence, high-protein ONS enriched with “HMB and vitamin D” seems to be a promising nutritional intervention to facilitate recovery of muscle mass decline and dysfunction in patients at risk of malnutrition and sarcopenia, particularly among geriatric patients with MCI or dementia (22, 140).

### Nutritional perspective beyond HMB and vitamin D—Multidomain interventions in cognitive aging

Besides the nutritional interventions specifically targeting muscle, many interventions have been gaining attention for their potential effects in preserving cognition through normalizing metabolic aberrations (i.e., increased levels of diet antioxidants- vitamins and carotenoids, colorful fruits and vegetables, such as berries, leafy greens, broccoli, tomatoes) and neuroprotection (i.e., intermittent fasting, folic acid and vitamin B12, omega-3 fatty acids and multidomain lifestyle interventions with Nordic diet or Mediterranean diet, physical exercise, cognitive training, social activities, and managing vascular and metabolic risk factors) (252, 256–260).

### Adjuvant exercise—Resistance exercise

Exercise interventions are considered the cornerstone of non-pharmacological sarcopenia management, with numerous studies demonstrating the robust muscle health benefits of resistance-based protocols, including improvements in muscle mass, strength, and physical function (261–263).

Resistance exercise, by modulating myokine release (BDNF, IGF-1, irisin, and cathepsin B) and mediating muscle brain crosstalk, has also emerged as a promising a multifaceted intervention capable of addressing both physical decline (activation of anabolic pathways) and cognitive decline (improvements in executive function, memory and global cognition) in older adults with MCI and sarcopenia (264).

Considering the adjuvant exercise, the potential synergistic effect of physical activity with nutritional support is emphasized in preventing age-related loss of muscle mass and strength (265, 266). While both exercise (resistance training in particular) and nutritional interventions (adequate protein intake, vitamin D, and other micronutrients in particular) serve as anabolic stimuli to skeletal myocytes, their combination has additive effects on long-term muscle health (20, 263, 267).

However, real-world evidence on combined application of muscle-targeted ONS and structured exercise remains limited and controversial (262). Some studies indicated that combining exercise with muscle targeted ONS (i.e., HMB and vitamin D) helps preserve muscle mass and physical function in older adults, emphasizing a synergistic interplay between resistance exercise and the HMB/vitamin D enriched ONS (262, 268, 269). Conversely, other studies showed that HMB plus vitamin D improves muscle function and lean body mass independently or alongside training, while do not provide added cognitive benefits over exercise alone in dementia or cognitive decline (164, 190, 270).

Therefore, the exercise regimen is considered likely to be the main driver of the observed improvements, while the nutritional supplementation to play a supportive role by optimizing substrate availability for muscle recovery and adaptation (262).

Noteworthy, individualized intervention strategies based on the patient's disease stage and functional status, rather than a one-size-fits-all prescription, are suggested to be more effective in managing patients with sarcopenia (263). Exercise response varies greatly depending on age, comorbidities, and physical functioning, and thus implementing standard exercise plans in immobile, severely frail, or advanced dementia patients is very challenging which requires a shift from active rehabilitation to supportive, palliative, and function-preserving strategies (263).

## Conclusion

In conclusion, the integration of the “malnutrition-sarcopenia-frailty” triad into nutritional care for dementia patients in clinical practice appears to be essential for ensuring continuity of dementia care, as preserving muscle health is vital for maintaining cognitive function in geriatric syndromes in the context of an active aging paradigm, shifting the focus of longevity from waiting for frailty to living with strength by placing value on additional years (7, 19, 21, 271). In this regard, given the interconnectedness between the vicious cycle of “malnutrition-sarcopenia-frailty” and the cognitive impairment through aging and shared mechanisms of muscle-brain crosstalk (7, 15), a high protein-ONS enriched with HMB plus vitamin D may be considered a convenient and compliant nutritional strategy to support muscle health and potentially contribute to preservation of cognitive health in older adults with or at risk of dementia, although direct evidence for cognitive benefit remains limited. Nonetheless, barriers to adoption of HMB and vitamin D fortified high protein ONS in dementia care should also be acknowledged, such as cost of specialized ONS, poor oral intake and swallowing difficulties among people with dementia, adherence challenges, variable access in long term care settings, and lack of unified international clinical guidelines specifically recommending these formulations for dementia cohorts, leading to considerable implementation gaps in routine care.
